# Supplementary material for: The effects of aging and an episodic specificity induction on spontaneous task-unrelated thought
Source: PLoS One. 2020 Aug 10;15(8):e0237340. doi: 10.1371/journal.pone.0237340 (PMC7416953; doi:10.1371/journal.pone.0237340)
Supplement: S1 File — (DOCX) [file pone.0237340.s001.docx]

The effects of aging and an episodic specificity induction on spontaneous task-unrelated thought

Magda Jordão^1*#^, Maria Salomé Pinho^1^, Peggy L. St. Jacques^2^

^1^Faculdade de Psicologia e de Ciências da Educação, Univ Coimbra, Portugal

^2^ Department of Psychology, University of Alberta, Canada

*Corresponding author, e-mail: [magda.jordao@gmail.com](mailto:magda.jordao@gmail.com)

^#^Current address: Faculdade de Psicologia e Ciências da Educação da Universidade de Coimbra, Rua do Colégio Novo, 3000-115, Coimbra, Portugal.

**Supplementary Material 1. Vigilance task - words and schematic representation**

Words presented in the vigilance tasks and descriptive summary of words’ valence, arousal (norms from Soares, Comesaña, Pinheiro, Simões, & Frade, 2012), and frequency, concreteness and imageability (norms from Soares, Costa, Machado, Comesaña, & Oliveira, 2017) by vigilance task

| **Vigilance task** | **European Portuguese** | **English** |
| --- | --- | --- |
| A | açúcar | sugar |
| A | alergia | allergy |
| A | ambulância | ambulance |
| A | áspero | harsh |
| A | assento | seat |
| A | autocarro | bus |
| A | avenida | avenue |
| A | bandeira | flag |
| A | bar | pub |
| A | beco | alley |
| A | beijo | kiss |
| A | brinquedo | toy |
| A | carruagem | wagon |
| A | casal | couple |
| A | casamento | wedding |
| A | casino | casino |
| A | centopeia | roach |
| A | chave | key |
| A | chuva | rain |
| A | coluna | column |
| A | comida | food |
| A | contente | glad |
| A | corredor | aisle |
| A | cozinheiro | cook |
| A | dentista | dentist |
| A | diabo | devil |
| A | diamante | diamond |
| A | diploma | diploma |
| A | doente | sick |
| A | escuro | dark |
| A | esposa | wife |
| A | faca | knife |
| A | famoso | famous |
| A | febre | fever |
| A | fogo | fire |
| A | forno | oven |
| A | germes | germs |
| A | gozo | enjoyment |
| A | igreja | church |
| A | infantário | nursery |
| A | jogo | game |
| A | lamacento | muddy |
| A | lâmpada | lightbulb |
| A | larva | maggot |
| A | leão | lion |
| A | lenço | handkerchief |
| A | ligadura | bandage |
| A | lixo | garbage |
| A | manteiga | butter |
| A | milionário | millionaire |
| A | mosquito | mosquito |
| A | motor | engine |
| A | mundo | world |
| A | nu | naked |
| A | nublado | overcast |
| A | peixe | fish |
| A | planta | plant |
| A | podre | rotten |
| A | pomba | dove |
| A | porco | pig |
| A | prenda | gift |
| A | provocador | defiant |
| A | quebrado | broken |
| A | relâmpago | lightning |
| A | salvamento | rescue |
| A | serpente | serpent |
| A | tecido | tissue |
| A | tesoura | scissors |
| A | torre | tower |
| A | trombeta | trumpet |
| A | urina | urine |
| A | vulcão | volcano |
| B | adulto | adult |
| B | agulha | needle |
| B | alerta | alert |
| B | aranha | spider |
| B | atleta | athlete |
| B | bebé | baby |
| B | bebida | drink |
| B | bengala | crutch |
| B | bolha | blister |
| B | bolor | mildew |
| B | brutal | brutal |
| B | cadeira | chair |
| B | calor | heat |
| B | cicatriz | scar |
| B | colete | vest |
| B | computador | computer |
| B | coração | heart |
| B | coroa | crown |
| B | criança | child |
| B | dinheiro | money |
| B | elevador | elevator |
| B | emprego | employment |
| B | escorpião | scorpion |
| B | escritório | office |
| B | falcão | hawk |
| B | feio | ugly |
| B | ferramenta | tool |
| B | ferro | iron |
| B | forte | strong |
| B | frio | cold |
| B | garrafa | bottle |
| B | homem | man |
| B | insecto | insect |
| B | irmão | brother |
| B | leite | milk |
| B | lesão | lesion |
| B | louco | mad |
| B | lucro | profit |
| B | máquina | machine |
| B | morgue | morgue |
| B | muco | mucus |
| B | muleta | crutch |
| B | musculado | muscular |
| B | namorado | sweetheart |
| B | natal | christmas |
| B | nó | knot |
| B | noiva | bride |
| B | obesidade | obesity |
| B | pântano | marsh |
| B | paralisia | paralysis |
| B | pecado | sin |
| B | petróleo | kerosene |
| B | porcaria | junk |
| B | rapaz | boy |
| B | relógio | clock |
| B | relvado | lawn |
| B | réptil | reptile |
| B | rocha | rock |
| B | rua | street |
| B | sarampo | measles |
| B | sujo | dirty |
| B | tabaco | tobacco |
| B | tanque | tank |
| B | táxi | taxi |
| B | termómetro | thermometer |
| B | troféu | trophy |
| B | tubarão | shark |
| B | veículo | vehicle |
| B | vermelho | red |
| B | vespa | wasp |
| B | vidro | glass |
| B | vinho | wine |

|  | Vigilance Task A  *N* = 72 | | Vigilance Task B  *N* = 72 | |
| --- | --- | --- | --- | --- |
|  | *M* | *SD* | *M* | *SD* |
| Valence | 5.10 | 1.31 | 5.07 | 1.38 |
| Arousal | 4.80 | .73 | 4.83 | .65 |
| Frequency | 26.20 | 69.31 | 28.39 | 52.62 |
| Concreteness | 5.70 | .94 | 5.75 | .96 |
| Imageability | 5.59 | .64 | 5.59 | .71 |

*Note:* Valence, arousal, concreteness and imageability were measured in scale increasing scale from 1 to 9. Frequency was given by frequency per million of occurrences in the linguistic corpus. There were no significant differences between the words in vigilance task A and B (all *p* > .721).

Schematic representation of the vigilance task

References

Soares, A. P., Comesaña, M., Pinheiro, A. P., Simões, A., & Frade, C. S. (2012). The adaptation of the affective norms for english words (ANEW) for european portuguese. *Behavior Research Methods, 44*(1), 256-269. doi: 10.3758/s13428-011-0131-7

Soares, A. P., Costa, A. S., Machado, J., Comesaña, M., & Oliveira, H. M. (2017). The minho word pool: Norms for imageability, concreteness, and subjective frequency for 3,800 portuguese words*. Behavior Research Methods, 49*, 1065–1081. doi: 10.3758/s13428-016-0767-4
