# Supplementary material for: The effects of aging and an episodic specificity induction on spontaneous task-unrelated thought
Source: PLoS One. 2020 Aug 10;15(8):e0237340. doi: 10.1371/journal.pone.0237340 (PMC7416953; doi:10.1371/journal.pone.0237340)
Supplement: S2 File — (DOCX) [file pone.0237340.s002.docx]

The effects of aging and an episodic specificity induction on spontaneous task-unrelated thought

Magda Jordão^1*#^, Maria Salomé Pinho^1^, Peggy L. St. Jacques^2^

^1^Faculdade de Psicologia e de Ciências da Educação, Univ Coimbra, Portugal

^2^ Department of Psychology, University of Alberta, Canada

*Corresponding author, e-mail: [magda.jordao@gmail.com](mailto:magda.jordao@gmail.com)

^#^Current address: Faculdade de Psicologia e Ciências da Educação da Universidade de Coimbra, Rua do Colégio Novo, 3000-115, Coimbra, Portugal.

**Supplementary Material 2. Coding instructions**

1. If the content includes one of the following situations, please select:
2. It is only a music (earworm);
3. It is only noticing something in the environment without further elaboration (external distraction);
4. It is only the repetition of the last word presented on the screen^[[1]](#footnote-1)^.
5. Is the **content** described by the participant related with the task? Please code:
6. The content is related to the task.
7. The content is not related with the task.

**Examples** of thoughts related with the task: “I was reminding myself to say yes when a yellow word appears”, “I was wondering if the words would repeat”, "The word *sour* brought up an aversion feeling", "I was thinking I was more concentrated now than at the beginning of the task".

1. For task-unrelated thoughts code episodic specificity from 0 to 4 within Piolino et al. (2006) guidelines.

**Notes to the episodic specificity coding**

- The time and place information may be explicit or implicit.
- General knowledge (facts about the world and public events) should be coded 0 even if they refer to facts that occur in certain time epoch and country. **However**, there may be cases in which general knowledge is represented in an image or scene with some detail, suggesting a higher level of episodic construction (e.g., “I saw many refugees in a small boat, struggling”, referring to knowledge about the refugee crisis) compared to simply stating general facts or images. These cases should be coded 1.
- When several events are mentioned by the participants and you are unsure which one is central, take into account what was the participant report about the predominant event (given in the temporality score). When the description includes general knowledge and/or personal semantics **and** an event, **the event should be the focus of coding**.
- The cognitive, perceptual and emotional details must refer to something experienced in the event described. This means that thoughts or emotion about recalling/imagining the event **now** are not considered (e.g. “This is silly, but I was remembering…).

**Reference**

Piolino, P., Desgranges, B., Clarys, D., Guillery-Girard, B., Taconnat, L., Isingrini, M., & Eustache, F. (2006). Autobiographical memory, autonoetic consciousness, and self-perspective in aging. *Psychology and Aging*, *21*(3), 510–525. https://doi.org/10.1037/0882-7974.21.3.510

1. These cases were flagged because it was not clear how they should be classified, given that the content is not about the task (as in e.g., “I was thinking the task is very slow”), but is only a repetition of an element presented in it, without any further elaboration. After discussion with a senior researcher, and given the absence of any task-unrelated information in these cases, we decided to classify them as task-related thoughts. [↑](#footnote-ref-1)
