# Supplementary material for: The effects of aging and an episodic specificity induction on spontaneous task-unrelated thought
Source: PLoS One. 2020 Aug 10;15(8):e0237340. doi: 10.1371/journal.pone.0237340 (PMC7416953; doi:10.1371/journal.pone.0237340)
Supplement: S3 File — (DOCX) [file pone.0237340.s003.docx]

The effects of aging and an episodic specificity induction on spontaneous task-unrelated thought

Magda Jordão^1*#^, Maria Salomé Pinho^1^, Peggy L. St. Jacques^2^

^1^ Faculdade de Psicologia e de Ciências da Educação, Univ Coimbra, Portugal

^2^ Department of Psychology, University of Alberta, Canada

*Corresponding author, e-mail: [magda.jordao@gmail.com](mailto:magda.jordao@gmail.com)

^#^Current address: Faculdade de Psicologia e Ciências da Educação da Universidade de Coimbra, Rua do Colégio Novo, 3000-115, Coimbra, Portugal.

**Supplementary Material 3. Descriptive statistics of sTUTs by trigger, temporality and verbal/visual form**

Table 1. *Mean frequency (standard deviation) of sTUTs in each trigger status (without, with trigger) by age group in the episodic specificity induction (ESI) and in the control induction.*

|  | Without trigger | | | With trigger | | |
| --- | --- | --- | --- | --- | --- | --- |
|  | Younger | Older | Younger | | Older |  |
| ESI | 0.63 (0.82) | 0.63 (0.97) | 3.96 (3.25) | | 3.67 (3.50) |  |
| Control | 0.79 (0.88) | 0.67 (1.09) | 4.00 (3.09) | | 3.67 (3.36) |  |
| Total | 0.71 (0.72) | 0.65 (0.72) | 3.98 (3.10) | | 3.67 (3.10) |  |

Table 2. *Mean frequency (standard deviation) of sTUTs in each temporality (past, present, future, atemporal) by age group in the episodic specificity induction (ESI) and in the control induction*^[[1]](#footnote-1)^*.*

|  | Past | | Present | | Future | | Atemporal | |
| --- | --- | --- | --- | --- | --- | --- | --- | --- |
|  | Younger | Older | Younger | Older | Younger | Older | Younger | Older |
| ESI | 1.83 (1.97) | 1.29 (1.68) | 0.33 (0.56) | 1.08 (1.18) | 0.83 (1.09) | 0.58 (1.25) | 1.58 (2.55) | 1.25 (1.92) |
| Control | 1.50 (1.82) | 1.63 (2.23) | 0.17 (0.38) | 1.17 (1.20) | 1.29 (1.43) | 0.46 (0.78) | 1.83 (2.12) | 0.83 (1.20) |
| Total | 1.67 (1.67) | 1.46 (1.67) | 0.25 (0.74) | 1.13 (0.74) | 1.06 (1.00) | 0.53 (1.00) | 1.71 (1.77) | 1.04 (1.77) |

Table 3. *Mean frequency (standard deviation) of sTUTs in each form (verbal, visual) by age group in the episodic specificity induction (ESI) and in the control induction.*

|  | Verbal | | | Visual | | |
| --- | --- | --- | --- | --- | --- | --- |
|  | Younger | Older | Younger | | Older |  |
| ESI | 1.29 (1.12) | 2.21 (1.86) | 3.29 (2.93) | | 2.04 (2.46) |  |
| Control | 1.71 (1.40) | 2.21 (2.04) | 3.08 (2.55) | | 1.96 (2.18) |  |
| Total | 1.50 (1.46) | 2.21 (1.46) | 3.19 (2.34) | | 2.00 (2.34) |  |

1. There were some instances in which participants reported more than one temporal-orientation for the thought described (e.g., thinking about a past experience that is going to happen in the future, or an ongoing/present situation that is going to have a future resolution…). These mixed temporality cases were only 1.85% of thoughts collected, and were not included in the temporality analysis. [↑](#footnote-ref-1)
