# Supplementary material for: The effects of aging and an episodic specificity induction on spontaneous task-unrelated thought
Source: PLoS One. 2020 Aug 10;15(8):e0237340. doi: 10.1371/journal.pone.0237340 (PMC7416953; doi:10.1371/journal.pone.0237340)
Supplement: Attachment — Submitted filename: Response to reviewers_Final.docx [file pone.0237340.s004.docx]

**Response to Reviewers**

**Reviewer #1**

Please note that the indications for lines in our replies refer to the manuscript with track changes

**Minor point
298-368: The main recommendation is a need for more clarity in the description of variables measured. Quite a large number of characteristics are measured based on participants report. Each measure is described in detail, however, because of their number and strong similarity for some, it would be important to add a small paragraph restating briefly the name and purpose of these dependent variables. A graph may be considered to help visualise the process by which these measures were computed. This should enable a better understanding of the study and the later outlined results.
R:** Thank you for this suggestion. We created a new figure to summarize and clarify the coding and resulting variables (Fig. 3), and added a paragraph that briefly restates the variables at the end of this section (lines 402-407).

**Other recommendations are as follow:
203: A threshold for BDI scores needs to be provided.**

**R:** We’ve now added the BDI threshold on lines 212-213.

**Additionally, it is not mentioned whether some participants have been excluded based on exclusion criteria.**

**R:** Thank you for noticing this. We’ve now added the information about the number of participants who were excluded based on the exclusion criteria, on lines 216-219.

**Presence or absence of any compensation for participants’ time and travel should be stated.**

**R:** This is also now added on lines 197-199.

**253: From reading the paper, it seems that information about how motivation was evaluated is missing.**

**R:** Thank you for highlighting this issue. We’ve now added information about the scale we used to measure motivation, on lines 276-277.

**In the same line (283), it is unclear whether questions were asked verbally by the experimenter or presented on the computer screen.**

**R:** We know clarify that questions were presented verbally by the experimenter on line 312.

**438: A word seems to be missing in this sentence.**

**R:** We’ve revised the sentence and added the missing word, on line 490.

**506: potentially related to the lack of clarity in the method section. It is difficult to see what is referred to by “the overall, strictly episodic scores, and episodic events with and without detail”.
R:** Following your suggestion for the methods, we’ve now added a figure (Fig 3) that summarizes what these measures represent and to make this clearer. We now refer back to the Fig 3 here (lines 566-567).

**540-454: This section needs to be reworded, potentially cutting the sentence, to help the reader fully capture key information.**
**R:** I believe the reviewer means 540-545. We’ve reworded this sentence now, cutting the sentence, as suggested (lines 601-605).

**565: Problems with table format.**

**R:** Thank you for letting us now you had problems with the table. We’ve now rearranged the table to better fit the manuscript page (line 627). For consistency in the layout of the variables (namely, age group and type of induction), we’ve also rearranged the other tables.

**567-594: In this part of the results section, analyses are conducted on overall specificity scores. However, results are reported only in terms of frequency (e.g. “past-oriented sTUTs were more frequent (M = 0.43, SD = 0.67) than future-oriented ones (M = 0.05, SD = 0.21)”). This is confusing, this section is supposed to evaluate episodic specificity in past and future thoughts, yet results mention that past thoughts are more frequent then future one without mention of episodic specificity. It is probably just a case of rewording to enable better understanding.**

**R:** Thank you for pointing this out. We now reword this section to improve clarity (section 3.6.3., starting on line 629).

**582: It is stated that all other results are p >.06. For completeness, authors may consider stating which effect is, to some extent, approaching significance. This is particularly important as elsewhere in the manuscript a marginal effect has been reported (p<.054). The information provided so far may bring questions regarding selection of information. To be more transparent and consistent, p-values close to .06 should be reported with more details.**

**R:** We now provide more information about the effect that is approaching significance on lines 645-648.

**SM2: It is unclear to which coding aspect the “Note” section is referring too.**
**R:** Thank you for highlighting this issue. In a revised version of the SM2, we now specify that the “Note” section refers to the episodic specificity coding.

**Reviewer #2**

Please note that the indications for lines in our replies refer to the manuscript with track changes

**MAJOR POINTS
The within subjects design allows authors to examine one very interesting question that was not addressed in the paper. Namely, it would be very interesting to see if the number of sTUTs correlates positively across the two sessions. This should be possible to do given that there was no significant effect of ESI. I realise that this is perhaps a minor issue for the authors, but I was wondering if it was possible to check this and report perhaps somewhere in the results or discussion section?**

**R:** Thank you for this interesting suggestion. We now include this analysis, that turned out to be a significant positive correlation, on lines 496-501.

**Method section
1. Lines 243-245 – Please provide more details about the episodic specificity induction procedure which will be useful to readers who are unfamiliar to it. For example, what are the instructions that participants receive? What exactly did the videos contain, are these videos comparable?**

**R:** We’ve now added some more details about the instructions and the content of the videos, on lines 262-264.

**2. Lines 253-270 – Section 2.5 describing the vigilance task was very difficult to understand. In fact I could not understand at all what were the target stimuli, how did the words appear on trials, what words were they, etc etc? Many sentences are unclear. For example, “ Prior to starting the task participants assessed their motivation level, to avoid the influence of perceived performance on these ratings (58)” (Can you please explain what did this involve?). OR “Our earlier study found no evidence of age differences in MW frequency (17), suggesting that the adaptation controlled for confounding factors, such as possible age-related differences in the use of response options (8)” (what does this mean? Can you explain what response option means here?). OR “Second, we controlled for the number and characteristics of the word cues presented between each probe. Third, we changed the ISI from 7 to 3 seconds, which was important because longer ISIs require spontaneous thoughts triggered by word-cues to be maintained longer to be caught in the probes. Thus, by reducing the ISI, we are able to record both longer and shorter thoughts”. OR “The final task took 15.9 minutes, and randomly presented 72 words and 87 five-point sequences for 3 seconds, followed by a 3 seconds ISI” .**

**It is unclear in these descriptions how many trials were used, what were target and non-target stimuli, how many trails had words and how many trials did not have any words on them, how were the words selected, did they differ in valence, etc. Did the vigilance task last for 15.9 minutes without the thought probes or with thought probes? How many trials were there between two consecutive probes? What do fife-point sequences refer to? Etc.**

**R:** Thank you for highlighting these issues. We’ve now tried to clarify our task following your suggestions. Below we address each on of the point raised about the vigilance task description.

**“ Prior to starting the task participants assessed their motivation level, to avoid the influence of perceived performance on these ratings (58)” (Can you please explain what did this involve?).**

**R:** We’ve now added information about the motivation level assessment and rephrased its purpose, on lines 275-278.

**OR “Our earlier study found no evidence of age differences in MW frequency (17), suggesting that the adaptation controlled for confounding factors, such as possible age-related differences in the use of response options (8)” (what does this mean? Can you explain what response option means here?)**

**R:** We’ve now clarified that response options refer to pre-determined response options, in a forced-choice format, used to categorize what was on the participant mind (as now added to the manuscript on lines 286-287).

**OR “Second, we controlled for the number and characteristics of the word cues presented between each probe. Third, we changed the ISI from 7 to 3 seconds, which was important because longer ISIs require spontaneous thoughts triggered by word-cues to be maintained longer to be caught in the probes. Thus, by reducing the ISI, we are able to record both longer and shorter thoughts”.**

**R:** We’ve now rephrased for clarity the controls that have been put in place between each probe and the rational for using a 3 seconds ISI. For the ISI we dropped the comparison with the previous task and instead tried to clarify how we thought it could benefit the present task (on lines 289-297).

**OR “The final task took 15.9 minutes, and randomly presented 72 words and 87 five-point sequences for 3 seconds, followed by a 3 seconds ISI” . It is unclear in these descriptions how many trials were used**

**R:** We’ve now clarified that the vigilance task included 159 trials (line 300).

… **what were target and non-target stimuli,**

**R:** We’ve now clarified that the stimuli presented in the color yellow were the target (line 303).

**…how many trails had words and how many trials did not have any words on them**

**R:** We’ve now clarified that we either presented words OR five-point sequences, so there were 72 words presented (lines 298-300). We also rephrased five-point sequences to five-dot sequences, which hopefully will be clearer, and added in brackets that we mean this stimulus . . . . . Finally, we referred to the visual representation of the task in the Supplementary Material 1.

**…how were the words selected, did they differ in valence, etc.**

**R**: The words were selected from a well-known word database (as now mentioned on lines 279-280) that provided data on valence, arousal, frequency, concreteness and imageability. We used these data to match the words selected for each version of the vigilance task. The words used and the statistical comparison between versions for each word characteristic is presented in the Supplementary Material 1, as we now mention in the text (line 281).

**Did the vigilance task last for 15.9 minutes without the thought probes or with thought probes?**

**R:** 15.9 minutes was the presentation time for the task, without the probes, as now added on line 298.

**How many trials were there between two consecutive probes?**

**R:** This was variable, in order to avoid making the thought probe predictable. There were 9, 13 or 18 trials between probes, as now added to the manuscript on line 305.

**What do fife-point sequences refer to? Etc.**

**R:** As mentioned above, these were sequences of five dots . . . . . that were presented interchangeably with the words. Please check lines 299-300, where we rephrased the expression from five-point sequences to five-dot sequences, and also the Supplementary Material 1 (S1 File), where this is shown in a schematic figure of the vigilance task.

**3. In section 2.6.1, please provide examples of task-unrelated and task-related thoughts provided by participants. On line 314-315 can you please provide some examples of external distracters, as the definition provided is a bit unclear and long-winded?**

**R:** We know present examples for task-related thoughts (on lines 339-340) and external distractions (on lines 346-347). We also rephrased the definition of external distractions (on lines 343-346). For task-unrelated thoughts we added a note to the Fig 2, which includes several examples of sTUTs (with different levels of specificity), on lines 340-341.

**4. In section 2.6.3 – please indicate who were the coders? Any of the authors or researchers helping to conduct the study?**

**R:** Thank you for your question. Several students at an undergraduate and graduate level collaborated in this research, as an optional credit or as part of their research assistant role duties. MJ was also a coder and that is also now clearly identified, along with a correction on the information about blinding – MJ was naturally not blind to the hypothesis. Thank you for highlighting this aspect and allowing us to make this correction. You can find this info now summarized on section 2.6.3, lines 414-421.

**Results**
**1. Section 3.3 – Can you please start this section by providing some descriptive information about a total number of thought probes obtained in 24 young and 24 old participants in ESI and Control conditions (i.e., 288 probes in each of the 4 conditions), an how many probes were “no thoughts”, “earworms”, “distractions” etc?** **This could then be followed up by means presented in Table 2. However, given that there were probably very few instances of earworms (as few as “no thoughts”) is it worth presenting earworm category in Table 2? It will be also useful to have some descriptive information on reports of thought triggers and if they were predominantly the cue words presented in trials preceding the thought probe?**

**R:** We’ve now added the number of overall probes (on line 455), and the number of probes in which spontaneous thought was reported by age group and type of thought (lines 458-461).

Regarding the presentation of the data about earworms, we believe it is important to include this information because we found a different pattern of age-related differences (younger adults experience more earworms). This is in line with previous research that we mention on lines 349-350 and suggests that “musical memories rely on a different cognitive system and show distinct age-related patterns”.

We’ve also added information about type of trigger, confirming they were predominantly the cue words presented in the task previously to the probe (lines 523-534).

**2. Lines 471-472 – It would be clearer if you stated that participants had significantly higher number of past than present thoughts, and it would be also useful to report comparisons and means for future thoughts.**

**R:** We rephrased the manuscript according to the suggestion (line 530). Here and throughout the manuscript we only detailed significant post-hoc comparison results, for the sake of simplicity. However, the detailed data for all temporal orientations, including means and SDs per group, per condition and overall, is presented in the Supplementary Material 3 (S3 File), as mentioned in the beginning of this section (lines 514-515).

**3. Section 3.6.3 – The 2 (age group: young, older) × 2 (type of induction: ESI, control) × 2 (temporality: past, future) mixed ANOVA on each of the specificity measures may potentially involve a loss of number of participants who did not report either past or future sTUTs. Can you please confirm that this was not the case? I.e., did all participants report at least one past and one future thought?
R:** Not all participants reported at least one past and one future thought. However, we did not exclude these participants from the analysis of specificity, because the specificity scores are based on the raw number of thoughts (of different levels of specificity, as now represented in Fig 3). Being so, when the participants did not have a past or future sTUT in one of the conditions, their score is simply zero, which is still a valid data point, and this participants’ data still feeds into the analysis. For example, if an older participant reports only past sTUTs, this will inform the analysis of the interaction between age group and temporality. Namely, this helps us to analyze if the age-related pattern of specificity in past-oriented thoughts is similar to the one found in the overall analysis.

We think it is also important to consider the disadvantages of excluding these participants. Unless we have reasons to believe that their data is for some reason unreliable, which we didn’t in our study, the fact that some participants do not report at least on past and future sTUT is just part of the natural inter-variability of this phenomenon. It would seem that not including them in the analysis would pose the risk of biasing our results and interpretations. Thus, it seems to us that the best option is to use a type of analysis that makes use of as much information as possible, which we try to by using frequencies as detailed above.

**General discussion
1. Lines 697-700 – It is pointed out that “Instead, spontaneous future thoughts would more appropriately be characterized as memories of future thoughts that have been deliberately recalled (and constructed) before. In fact, previous research supports the view that spontaneous future thoughts are “prestored representations of previously imagined events” (38, p. 269). In relation to this idea, it would be very highly relevant to mention or discuss the dual-processes account of future thinking proposed by Cole and Kvavilashvili (2020) which develops this idea into a coherent theoretical approach and reviews supporting empirical evidence**.

**R:** Thank you for mentioning this relevant reference. We’ve now added it to the manuscript (lines 766-768).

**Minor Points
1. Please use past tense in the abstract**
**R:** We’ve adapted the abstract accordingly (line 28).

**2. Line 62 – Please define what is episodic specificity immediately after using this term.**
**R:** We’ve now added the definition on lines 62-64.

**3. Line 81 – you could mention here two naturalistic studies by Warden et al. who consistently showed no age effects on involuntary past and future thoughts using diary and experience sampling methods? A study by Gardner and Ascoli (2015; Psychology and Aging) is also highly relevant.**

**R:** Thank you for mentioning these relevant references. We’ve now added these to the manuscript (lines 83-84).

**4. Lines 159-160 – It is pointed out that “Alternatively, it has been suggested that direct retrieval processes that characterize spontaneous thought imply the existence of pre-stored event representations, independent of event construction both for autobiographical recall and for episodic future thinking”. This is an important statement and it might be useful to slightly expand on this idea by citing studies of Jeunehomme and D’Argembeau (2016) and Cole and Kvavilahsvili (2020, in press in Psychological Research)?
R:** We’ve now expanded this idea including the references suggested (line 163-168).

**5. Lines 208- provide age ranges (what was the minimum and maximum age) in each group**
**R:** We’ve now added this information to the manuscript (lines 220-221).

**6. Lines 214-216 in Design – what were your main DVs?
R:** We’ve now added this information to the manuscript (lines 230-233).

**7. Line 220- Should be “Both sessions included an initial induction procedure (episodic or control), in which a video was presented, followed by a vigilance task to elicit spontaneous thoughts”.**
**R:** Thank you for the correction, now added to the manuscript (lines 237-238).

**8. Line 224 – Should be “There was an exception”**

**R:** Thank you for the correction, now added to the manuscript (line 241).

**9. Lines 445 and 449 – should be “sTUTs out of all spontaneous thoughts”
R:** Thank you for the correction, now added to the manuscript (lines 503 and 507).

**10. Line 506 – it would be helpful to refer readers to Fig 2 and define in brackets what each category refers to (i.e., scores >1, scores = 4, and scores 3 and 4, respectively)**

**R:** Following a suggestion of Reviewer 1, we’ve now created a new figure (Fig 3) that represents which events are included in each episodic measure. We’re referring to the Fig 3 as a reminder of what each of the measures mean (lines 566-567).

**11. Line 528 – “that inducing a targeting event construction does not” – this may need some re-wording?
R:** Yes, this should be “an induction targeting event construction”, thank you for pointing this out (now corrected on line 589).

**12. Lines 555 and 668 – I think referring to marginal findings as “anecdotal” is incorrect. You need to refer to them as “marginally significant”
R:** Thank you, this is now corrected (lines 616 and 733-734).

**13. Line 650 – should be “find age-related differences”**
**R:** Thank you, this is now corrected (lines 716).

**Figure 1 – instead of Elicitation task may be you should say Vigilance task?**

**R:** Thank you, this is now corrected in the new version of Fig 1.
