# Supplementary material for: The effects of aging and an episodic specificity induction on spontaneous task-unrelated thought
Source: PLoS One. 2020 Aug 10;15(8):e0237340. doi: 10.1371/journal.pone.0237340 (PMC7416953; doi:10.1371/journal.pone.0237340)
Supplement: Attachment — Submitted filename: Response to Reviewers.docx [file pone.0237340.s005.docx]

**Response to Reviewers**

**Reviewer #2**

Please note that the indications for lines in our replies refer to the manuscript with track changes

**Overall, the revision reads very well and the authors have tried to address most of my comments and suggestions, except the one on the vigilance task. Despite the additions made, the description of the vigilance task in Section 2.5 is still very confusing and lacks structure. The reader does not know anything about this task at this point, so it is essential to first introduce the vigilance task per se in terms of the number slides in total and which slides are targets and which slides are not targets. In other words, what is the main thing that participants have to do in this task? Also, I could not understand what ISI referred to. For how long was each slide presented and what was the interval (if any) between the two consecutive slides? Once this is all explained, then the authors could add information about the cue words and explain also why these words are added (i.e., to induce task unrelated thoughts in participants). After this, the authors could explain everything in relation to thought probes. In fact, information about thought probes could precede the information about the cue words, if the authors think that this is a better way of presenting this information. But in any case, the task needs to be described step by step, in a logically structured way, so that the reader can immediately grasp the key aspects of the study instead of getting more and more confused and stopping at the end of each sentence.**

**R:** We’ve now restructured the presentation of the task according to the suggestion (section 2.5, lines 267-349). After a brief mention of the motivation rating, that precedes the vigilance task, we describe what type and how many stimuli were presented, how long each were presented for, and what participants were asked to do. Then, we present the information related to the thought probes. After this, we add information about the cue words, including a mention to its role in inducing spontaneously retrieved thoughts. In the final paragraph of this section, we complement this information by providing additional detail about the reasoning behind the described features of the task.

**The second relatively minor point concerns the paragraph on correlational analysis on sTUTs frequencies at Time 1 and Time 2. This paragraph I think should be presented as the last paragraph in this section starting with “Finally, the within-participants design allowed us…”. It was encouraging to see that there was such a strong correlation between the frequencies. However, can the authors please present the correlations separately for young and older adults to ensure that this is not a spurious correlation?**

**R:** As suggested, we now presented this information as the last paragraph of the section and added the correlation information separately for younger and older adults, confirming that the correlation remains positive and significant for both groups (lines 528-533).

**Some other minor points:**

**Line 304 – Reference number is 364. This can not be correct?**

**R:** The reference number is 36, but in the track changes document this is not visible because the erased sign goes over the 4. This is visible in the previous manuscript without track changes (line 293) and in the present manuscript with track changes (line 281).

**Line 341 – Please delete “given**

**R:** Now deleted.

**Line 471 – Please ensure that title of Table 2 indicates that means refer to spontaneous thoughts**

**R:** Now added (line 489).

**Line 768 – Should be “and result from the activation of representations of previously constructed events”**

**R:** Yes, now corrected accordingly (line 785).
